# Increasing colorectal cancer screening among Alaska Native peoples living in remote areas of Alaska: a multitarget stool DNA cluster randomized controlled trial

**DOI:** 10.1186/s12875-026-03370-x

**Published:** 2026-05-26

**Authors:** Diana Redwood, Christie Flanagan, Lauren Jeffries, Peter Holck, Lila Finney Rutten, Christine Desnoyers, Danika Bailie, John Kisiel

**Affiliations:** 1 Alaska Native Tribal Health Consortium, 4000 Ambassador Drive, Anchorage, AK 99508 USA; 2 Holck Consulting, 1801 Norwood Ave, Boulder, CO 80304 USA; 3 Mayo Clinic, 200 1st St SW, Rochester, MN 55905 USA; 4 Yukon-Kuskokwim Health Corporation, 829 Chief Eddy Hoffman Hwy, Bethel, AK 99559 USA

**Keywords:** Alaska Native, Colorectal neoplasms, Screening, Early detection, Diagnosis, Stool tests, Randomized clinical trial

## Abstract

**Background:**

Increased colorectal cancer (CRC) screening is needed to address high Alaska Native (AN) CRC rates. The study objective was to evaluate high-intensity vs. medium-intensity patient outreach using multi-target stool DNA (mt-sDNA) or colonoscopy.

**Methods:**

A community-level, cluster-randomized clinical trial (April 29, 2021, through September 15, 2024) in 46 rural/remote Alaska communities. Participants were asymptomatic AN adults aged 45–75 with no family or personal history of CRC who were due for screening. High-intensity: Up to six patient navigator telephone calls with follow-up mailer. Medium-intensity: Mailed letter with one follow-up call. Usual care: Standard of care clinical visits. The primary outcome was screening completion within 12 months. Secondary outcomes included test preference, abnormal mt-sDNA follow-up colonoscopy, and colonoscopy outcomes.

**Results:**

A total of 2029 AN people (mean [SD] age, 56 [8.4] years; 816 women [40%]) were randomized including 587 to usual care, 696 to medium-intensity, and 746 to the high-intensity arm. Both men and women were significantly more likely to request mt-sDNA (70%) over colonoscopy (30%, *p* < 0.01), with participants ≥ 65 years old most likely to request mt-sDNA.

Screening completion within 12 months among the total population was significantly higher in the medium-intensity (24%) and high-intensity arms (28%) compared with usual care (6%) (p<0.01). Compared to usual care, CRC screening odds were higher with high-intensity (odds ratio [OR], 5.90; 95% CI, 3.86-9.04) and medium-intensity intervention (OR, 4.87; 95% CI, 3.18-7.46). Among those who agreed to screen, completion was similar in both intervention arms. Screening completion was 16% higher among high-intensity vs. medium-intensity participants (OR, 1.25; 95% CI, 0.91-1.71, p=0.19). Of the total eligible population, 254/2029 people (12.5%) had valid mt-sDNA tests and 278/2029 (13.7%) had complete colonoscopies. CRC/advanced neoplasia was found in over 25% of initial screening colonoscopies and 59% of colonoscopies performed after abnormal mt-sDNA.

**Conclusions:**

Both a medium- and a high-intensity intervention were significantly more effective at increasing screening initiation and completion rates compared with usual care in rural/remote Alaskan communities. Participants significantly preferred mt-sDNA for screening. These findings highlight the clinical utility of focused outreach strategies in primary care settings and support the use of mt-sDNA as a viable alternative to colonoscopy in geographically isolated populations.

**Trial registration:**

The study is registered with the Clinical Trials Registry (Stool DNA to Improve Colorectal Cancer Screening Among Alaska Native People, NCT04336397, https//www.cancer.gov/research/participate/clinicaltrialssearch/v? id=NCI202108691, registration date 04/03/2020) and was designed in accordance with the Consolidated Standards of Reporting Trials (CONSORT) statement.

**Supplementary Information:**

The online version contains supplementary material available at 10.1186/s12875-026-03370-x.

## Institutional review board statement

The study was conducted according to the guidelines of the Declaration of Helsinki, and approved by the Alaska Area Institutional Review Board (IRB #2019-04-038; 29 August 2019) and the tribal research review committees of the Alaska Native Tribal Health Consortium, Southcentral Foundation Board of Directors and the Yukon-Kuskokwim Health Corporation.

## Background

Preventing colorectal cancer (CRC) is an important health priority of Alaska Native Tribal health leaders and communities. Screening can prevent CRC through the identification and removal of colorectal polyps before they develop into cancer [1]. While CRC incidence and mortality rates have been declining in the U.S. since the 1980s, attributed largely to screening [1, 2], Alaska Native CRC rates remain the highest in the world [3]. There is a critical need for increased CRC screening in this population, which varies statewide from around 54% to 72% [4], however, traditional methods like colonoscopy are challenging as more than half of the Alaska Native population resides in widely distributed and remote road-less regions [5]. The fecal immunochemical stool test has started to be available but has not yet been widely adopted in the Alaska Tribal Health System, with an analysis of 2009–2015 data reporting that 97% of CRC screening tests were colonoscopies [6]. The home-based multi-target stool DNA test (mt-sDNA; Cologuard^®^, Exact Sciences Laboratories, Madison, WI) detects both blood and DNA markers of neoplasia and has been established as an effective alternative to other CRC screening test options [7, 8], but has not been evaluated for patient acceptability and uptake in the Alaska Native population. There is a critical need to test CRC prevention and screening methods that have high sensitivity for cancer, are acceptable to patients and providers, and are feasible to implement in primary care settings for long-term sustainability of screening efforts.

Both patient navigation and mailed health education materials are effective interventions to increase cancer screening adherence [9–14] but have not been systematically investigated for Alaska Native populations living in rural/remote areas. We evaluated the following hypotheses: (1) compared with usual care, high-intensity patient navigation will increase CRC screening rates by at least 20%; (2) compared with usual care, medium-intensity mailed outreach will increase CRC screening rates by at least 10%; and (3) more participants will prefer and complete mt-sDNA as a screening method compared with colonoscopy.

## Methods

### Ethics

The study (Clinical Trials Registry NCT04336397) was approved as minimal risk by the Alaska Area Institutional Review Board (AAIRB protocol #2019-04-038) and was designed and reported in accordance with the Consolidated Standards of Reporting Trials (CONSORT) statement and checklist. The study received a waiver of informed consent from the AAIRB as all screening age-eligible were offered the intervention in each community. The Alaska Native Tribal Health Consortium’s and Southcentral Foundation’s Ethics and Compliance Departments provided privacy consultations and approval for the waiver of consent. The study was tribally approved by the Alaska Native Tribal Health Consortium’s, Southcentral Foundation’s, and the Yukon-Kuskokwim Health Corporation’s Boards of Directors who also approved this manuscript.

### Eligibility and intervention

The study protocol, methods, and design have been described elsewhere [15]. Briefly, self-identified Alaska Native adults aged 45–75 with no family or personal history of CRC due for CRC screening were eligible if they had at least one tribal clinic visit in the past three years. Communities were cluster-randomized by the study biostatistician at the start of the intervention conducted between April 29, 2021, and February 9, 2023. Study staff had no access to the random allocation sequence and participants were blinded to intervention assignment. The three intervention arms included: (1) high-intensity telephone patient navigation (up to 6 calls) and one mailed letter [16 communities]; (2) medium-intensity intervention with one mailed letter and one follow-up phone call [16 communities]; and (3) usual clinical standard of care screening recommendations [14 communities]. People in the usual care communities did not receive any intervention outreach but did receive standard of care which included recommendations for screening by colonoscopy or FIT if they came into the clinic for other medical reasons and the provider noticed though chart review that they were age-eligible and were not up to date with screening. Participants in the high- and medium-intensity intervention arms were offered mt-sDNA as a screening test option in addition to colonoscopy.

Respondents that refused screening were noted in the study database and received no further follow-up. If they agreed to screening, intervention participants were offered the choice of mt-sDNA or colonoscopy using an outreach script adapted from the Exact Sciences Cologuard patient navigation script. Using the script, the patient navigator introduced themselves and that they were calling from the regional tribal health organization. The patient navigator went on to say that the patient’s health records indicated that they were due for colorectal cancer screening. The navigator let the patient know that the tribal health organization was starting a project to increase colorectal cancer screening, including trying out a new at-home screening test to see what people would think about it. The navigator then gave a short description of the mt-sDNA test as well as colonoscopy and asked which test the patient preferred to use. The patient navigator had additional scripted information on both mt-sDNA and colonoscopy if people wanted more information to compare the two tests. Participants in the medium-intensity arm received the same comparison information in a mailed screening options brochure. Phone calls were made at different times of the day, including evenings, to try to reach participants. The outreach script included a reminder that the participant was due for screening, education about the screening tests available (mt-sDNA or colonoscopy) and facilitation for overcoming barriers. All participants received the same follow-up after choosing a screening method regardless of intervention arm. Participants who agreed to screening with mt-sDNA were provided with mt-sDNA kits directly and no Exact Sciences navigation services were used during the study, although the call schedule after an individual had been sent a mt-sDNA kit was based on the Exact Sciences follow-up schedule, with up to five contact attempts within 30 days. In the study, outreach continued if the participant needed additional assistance. Participants who completed mt-sDNA but their sample wasn’t valid for processing were offered and mailed another kit for completion (up to four kits total).

The age range and risk criteria were based on mt-sDNA testing requirements; persons already adherent to screening guidelines (colonoscopy within 10 years, sigmoidoscopy within five years, or fecal occult blood test within preceding 12 months) were excluded. The Reach Effectiveness Adoption Implementation Maintenance (RE-AIM) Framework [16] was used to inform evaluation of individual-level moderators of test preference, screening adherence, and diagnostic follow-up, including differences by age and sex. Because RCTs for research among the Alaska Native population are rare, we employed a community-based participatory research approach [17–20] to incorporate the strength of Indigenous experts throughout the research process including study design, implementation, and interpretation and dissemination phases [15].

### Research setting

The trial was conducted in the Yukon-Kuskokwim Delta region in southwest Alaska [21]. Healthcare services are provided by the Yukon-Kuskokwim Health Corporation, which manages the regional hospital in the hub community of Bethel (population 6,300) and health clinics in 46 surrounding communities (populations ~ 50 to 1,300) with no road connection to the rest of the state. The region has 58 federally recognized tribes, primarily Yup’ik, Cup’ik, and Athabascan ethnicity and is similar in size to the state of Oregon. Colonoscopy requires travel to Bethel by boat, snowmobile, or small airplane.

### Data elements

Study data were collected and managed using REDCap (Research Electronic Data Capture) [22]. The primary outcome measure was CRC screening completion [complete colonoscopy; mt-sDNA with a negative result; or mt-sDNA with an abnormal result followed by diagnostic colonoscopy] in the medical record within 12 or 18 months of follow-up after randomization and intervention. Medical records were reviewed for screening completion by a single abstractor.

Secondary outcome measures included preference for mt-sDNA vs. colonoscopy, completion rates for mt-sDNA vs. colonoscopy, the proportion of people who received a follow-up colonoscopy after an abnormal mt-sDNA test result, and final test outcome. Histopathological results from biopsied tissue or excised lesions from complete colonoscopies were categorized based on the most clinically significant lesion present [23].

### Sample size and power

The sample size allocation for this study was based on a pre-specified ability to detect an expected increase of ≥ 10% in screening participation for usual care vs. any intervention as well as an increase of ≥ 10% for high-intensity vs. medium-intensity outreach. Given these expected screening rate improvements, and an expected non-intervention screen rate of 59% [24], we anticipated having more than 80% power to detect a statistically significant difference between the medium- and high-intensity interventions, at alpha of 0.05 with a sample of *n* = 616 participants in each of the three study arms.

### Statistical analysis

Communities were the unit of randomization and inference, with individuals nested within communities. Communities assigned to high- and medium-intensity interventions were randomized within matched community pairs based on pre-specified community-level characteristics (coastal versus non-coastal location, 2018 community population size, and census area). Control communities were not part of the matched-pair randomization. Consequently, comparisons between high and medium intervention arms respect the matched-pair design, whereas analyses involving control communities are unpaired. All analyses were conducted at the individual level, with clustering by community accounted for in variance estimation.

Analyses were performed separately for each binary screening outcome using outcome-specific analytic datasets. Two analytic configurations were used: (1) intervention-only analyses restricted to high- and medium-intensity communities, and (2) all-arms analyses including control, medium-, and high-intensity communities. Sex-stratified analyses were conducted by applying the same analytic procedures to sex-specific subsets of the data. The study took a pragmatic approach in which all analyses were intention-to-screen to compare screening outcomes regardless of the actual treatment received [25].

For each outcome and study arm, we reported the number of eligible individuals, the number experiencing the outcome, and the crude outcome proportion. Crude proportions are descriptive and are presented to summarize observed screening uptake. Primary inferential estimands were odds ratios (ORs) comparing intervention groups, reported with 95% confidence intervals and p-values based on cluster-robust inference when feasible.

For outcomes analyzed among intervention communities only, the primary comparison between high- and medium-intensity interventions used generalized estimating equations (GEE) with a logistic link. Models included intervention intensity and matched-pair indicators to respect the randomization scheme. An independence working correlation structure was specified, and inference was based on cluster-robust variance estimation with CR2 small-sample correction and Satterthwaite degrees of freedom, using communities as the clustering unit. The high versus medium intervention contrast is reported as an odds ratio with corresponding confidence interval and CR2-corrected p-value.

When paired GEE models were infeasible due to sparse data, rarity of some outcomes, or numerical instability, we attempted a secondary matched-pair fixed-effects logistic regression using the same robust variance estimation. If adjusted paired models remained infeasible, crude comparisons were reported and explicitly labeled as such.

When analyses included control communities, intervention effects were evaluated using unpaired logistic regression models with intervention arm as a categorical predictor. Because control communities were not matched, these models did not include pair indicators. However, models were adjusted for community-level characteristics used in the matching process. Population-averaged inference was obtained using cluster-robust (CR2) standard errors with Satterthwaite degrees of freedom. Odds ratios comparing medium versus control and high versus control, with confidence intervals and p-values, are reported. An omnibus Wald test of the joint null hypothesis that both intervention coefficients equal zero was used to assess overall differences across arms. All tests were two-sided and results with *p* < 0.05 were considered statistically significant. We performed all analyses using R version 4.4 [26].

## Results

### Randomization assignment

**Fig. 1:**
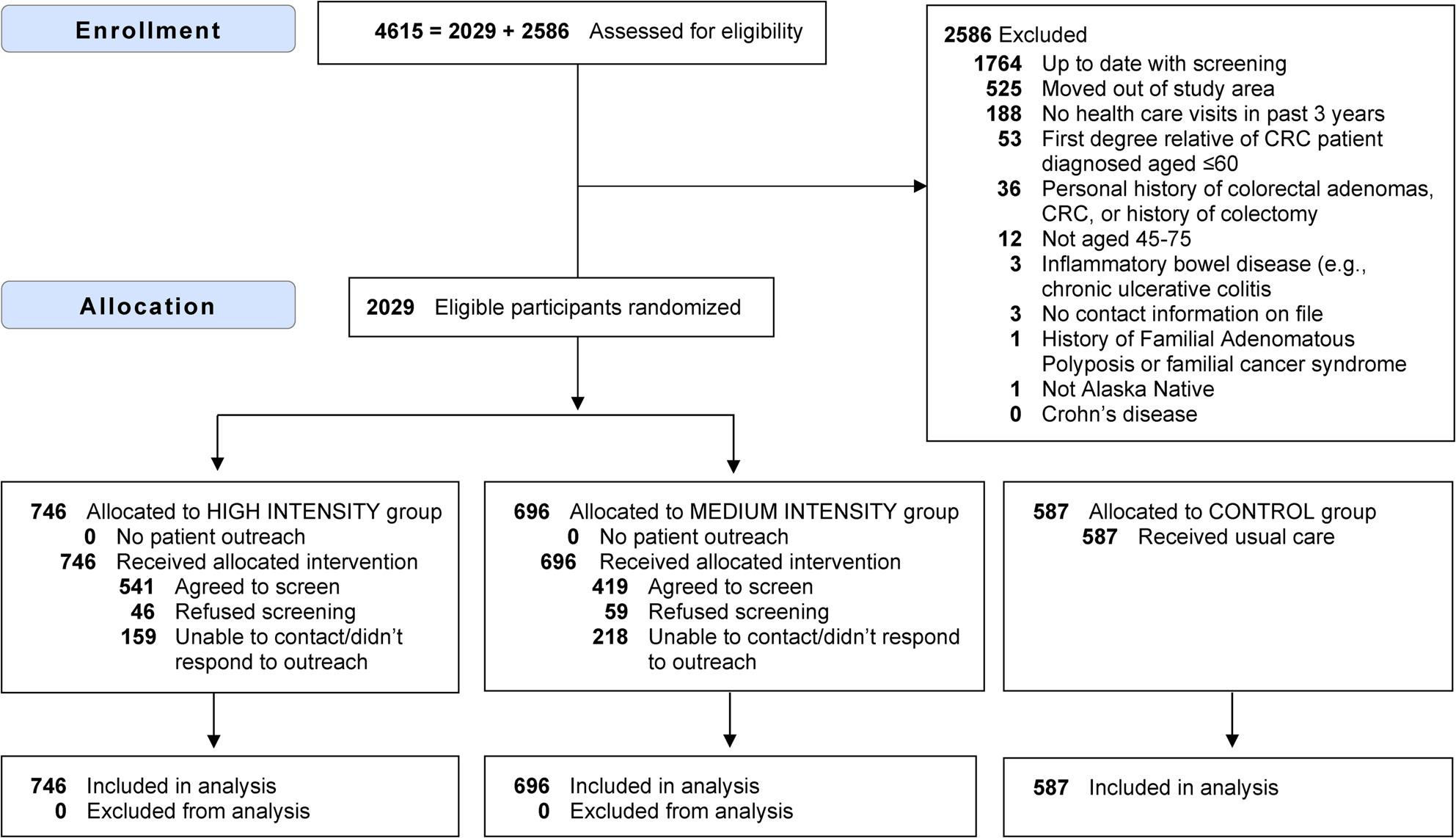
CONSORT Trial Flow Diagram. Explanatory figure legend: This diagram outlines the flow of participants through each stage of the randomized clinical trial in accordance with CONSORT guidelines. It presents the number of individuals assessed for eligibility, reasons for exclusion, and the total number randomized. For each study arm, the figure shows the number of participants allocated to the intervention, who received the assigned intervention, and who were excluded from analysis.

All Alaska Native adults aged 45–75 with a regional tribal medical record were assessed for eligibility (*n* = 4615) (Fig. 1). Of the 2586 (56%) excluded, the majority (68%) were already screened; a population baseline screening rate of 38%. An additional 20% were excluded because they had moved out of the region, 7% had no healthcare visits within three years or contact information on file, and 5% had a personal or family history of CRC in a first degree relative diagnosed ≤ 60 years old or another condition that made them ineligible for mt-sDNA testing. No eligble participants were excluded or considered lost to follow-up in the analysis (Fig. 1).

A total of 2029 Alaska Native adults (mean [SD] age, 55.9 [8.4] years; 816 women [40%]) were eligible and enrolled: 587 randomized to usual care, 696 randomized to the medium-intensity arm, and 746 randomized to the high-intensity arm (Fig. 1). Demographic covariates appeared reasonably balanced between study arms (Table 1).

**Table 1:** Baseline characteristics, colorectal cancer screening test requested, and test completed, by intervention arm

	Participants, *n* (%)				
	Total (*n* = 2029)	Usual Care (*n* = 587)	Medium Intensity (*n* = 696)	High Intensity (*n* = 746)	*P* value
**Baseline characteristic**					
**Age**					
Mean (SD), in years	55.9 (8.4)	55.0 (8.4)	56.4 (8.3)	56.2 (8.5)	
**Age group**					
45–54 years	970/2029 (47.8)	311/970 (32.1)	321/970 (33.1)	338/970 (34.8)	
55–64 years	687/2029 (33.9)	178/687 (25.9)	242/687 (35.2)	267/687 (38.9)	
65–75 years	372/2029 (18.3)	98/372 (26.3)	133/372 (35.8)	141/372 (37.9)	
**Sex**					
Women	816/2029 (40.2)	239/816 (29.3)	274/816 (33.6)	303/816 (37.1)	
Men	1213/2029 (59.8)	348/1213 (28.7)	422/1213 (34.8)	443/1213 (36.5)	
**Screening test requested**					
mt-sDNA	661/949 (69.7)	NA	277/410 (67.6)	384/539 (71.2)	< 0.01
Colonoscopy	288/949 (30.3)	NA	133/410 (32.4)	155/539 (28.8)	
**Screening test completed**					
mt-sDNA	254/2029 (12.5)	0/587 (0.0)	100/696 (14.4)	154/746 (20.6)	< 0.01
Colonoscopy (not including after abnormal mt-sDNA)	278/2029 (13.7)	38/587 (6.5)	118/696 (17.0)	122/746 (16.4)	
FIT	48/2029 (2.4)	19/587 (3.2)	15/696 (2.2)	14/746 (1.9)	
**Screening completion**					
Screening completion within 12 months among total study population	401/2029 (19.8)	32/587 (5.5)	164/696 (23.6)	205/746 (27.5)	< 0.01
Screening completion within 12 months among intervention participants who agreed to screen	351/960 (36.6)	NA	155/419 (37.0)	196/541 (36.2)	0.80
Mt-sDNA completion among those that agreed to screening and had a valid sample	254/661 (38.5)	NA	100/277 (36.2)	154/384 (40.2)	0.42
Mt-sDNA completion within 12 months among total mt-sDNA tests completed	250/254 (98.4)	NA	99/100 (99.0)	151/154 (98.1)	~ 1.00
Colonoscopy completion among those that agreed to screening and had a test performed	152/286 (53.1)	NA	67/131 (51.1)	85/155 (54.8)	0.56
Colonoscopy completion within 12 months among total colonoscopies completed	152/328 (46.3)	21/38 (55.3)	64/138 (46.4)	67/152 (44.1)	0.68

*Abbreviations*: *FIT *Fecal immunochemical test, *mt-sDNA *multi-target stool DNA, *NA *Not applicable, *SD *Standard deviation

Column percentages are provided for totals to allow comparison between study characteristics. Row percentages are provided for intervention arms (usual care, medium intensity and high intensity) to allow comparisons across intervention groups

### Outreach response

Among medium-intensity intervention participants, 419/696 (60%) agreed to screening, 59/696 (9%) explicitly refused screening, and 218/696 (31%) were unable to be contacted or didn’t respond to outreach. Among high-intensity arm participants, 541/769 (73%) agreed to screening, 46/746 (6%) refused screening, and 159/746 (21%) were unable to be contacted/didn’t respond to outreach (Fig. 1).

### Test preference

A greater percentage of participants in the medium-intensity arm (277/949; 29%) requested mt-sDNA than requested colonoscopy (133/949; 14%). This trend also held for the high-intensity arm, in which 384/949 (40%) requested mt-sDNA and 155/949 (16%) requested colonoscopy (Table 1). Men and women in both the medium-intensity (277/410; 68%) and the high-intensity arms (384/539; 71%) significantly preferred mt-sDNA for screening over colonoscopy (*p* < 0.01). Older (≥ 65 years) individuals were more likely to prefer mt-sDNA compared with the 45–54-year-old group (79% vs. 64%) (eTable 1 in the Supplement).

### Screening completion

Of the 2029 people enrolled, 254 (13%) were screened using mt-sDNA, 278 (14%) were screened using colonoscopy, and 48 (2%) were screened by fecal immunochemical test outside of the intervention (Table 1). During the 12-month intervention period, 401/2029 people (20%) in the total study population got screened by any test overall; 32/587 (6%) in the usual care arm, 164/696 (24%) in the medium-intensity arm, and 205/746 (28%) in the high-intensity arm. Among intervention participants who completed screening, 351/960 (37%) were screened within 12 months. This did not vary significantly by intervention arm: 155/419 (37%) in the medium-intensity and 196/541 (36%) in the high-intensity arm (*p* = 0.8) (Table 1).

Of the 661 participants who requested mt-sDNA and agreed to screening, 336 (51%) returned their mt-sDNA kit for processing. A total of 254 participants (39% of those who originally agreed to screening) had a valid sample, 100/277 (36%) in the medium-intensity arm and 154/384 (40%) in the high-intensity arm. Most mt-sDNA tests (250/254; 98%) were completed within 12 months. Mt-sDNA completion was not significantly higher for the high-intensity arm (151/154; 98%) compared with the medium-intensity arm (99/100; 99%) (p approaching 1.0) (Table 1).

Of the 286 participants that agreed to screening and requested colonoscopy for their screening method, 152/286 (53%) completed a colonoscopy, 85/155 (55%) from the high-intensity arm and 67/131 (51%) in the medium-intensity arm. Colonoscopy completion within 12 months was not significantly different between the high-intensity (67/152; 44%) and the medium-intensity arm (64/138; 46%) (*p* = 0.78) (Table 1). Because the study occurred during the COVID-19 pandemic, with elective surgery delays, we also examined colonoscopy completion rates within 18 months (72%) (eTable 2 in the Supplement). Of the total screening tests completed, older participants aged 65–75 were most likely to complete mt-sDNA (50%) while younger participants aged 45–54 were most likely to complete colonoscopy (49%). Men were more likely to complete mt-sDNA tests (50%) whereas women were more likely to complete colonoscopies (48%) (eTable 3 in the Supplement).

The odds of CRC screening were significantly higher for the high-intensity (odds ratio [OR], 5.90; 95% CI, 3.86–9.04) and medium-intensity arm (OR, 4.87; 95% CI, 3.18–7.46) compared with usual care (Table 2). The high-intensity arm had a non-significantly higher OR compared with the medium-intensity arm (OR, 1.25, 95% CI, 0.91–1.71). When stratified by sex, odds of getting screened in the high-intensity arm compared with usual care were higher for men (OR, 5.46; 95% CI 3.30–9.05) as well as women (OR, 6.68; 95% CI 4.02–11.1); similar results were found for men (OR, 4.75; 95% CI 2.64–8.54) and women (OR, 5.22; 95% CI 2.95–9.24) in the medium-intensity arm compared with usual care. The number of screens needed to detect one CRC or advanced neoplasm was 49.3 overall; 50.4 with mt-sDNA and 48.2 with colonoscopy (data not shown).

**Table 2:** Odds ratios of colorectal cancer screening receipt by intervention group, overall, and by sex

CRC screening completion	OR (95% CI)		
	Usual care	Medium Intensity	High Intensity
Overall	Reference	4.87 (3.18–7.46)	5.90 (3.86–9.04)
Sex			
Women	Reference	5.22 (2.95–9.24)	6.68 (4.02–11.1)
Men	Reference	4.75 (2.64–8.54)	5.46 (3.30–9.05)
CRC screening completion High vs. Medium	NA	Reference	1.25 (0.91–1.71)

### Screening test outcomes

A total of 57/254 (22%) participants had abnormal mt-sDNA results, of whom 49/57 (86%) completed a follow-up colonoscopy (Table 3). Of the 8/57 (14%) people with abnormal mt-sDNA tests who didn’t complete their colonoscopies, 3/8 (38%) completed their procedure after 18 months, 3/8 (38%) were scheduled for colonoscopy but didn’t complete the procedure, 1/8 (13%) had an incomplete colonoscopy, and 1/8 (13%) needed additional medical clearance before colonoscopy could be performed (data not shown).

**Table 3:** Colonoscopy outcomes after abnormal multi-target stool DNA vs. colonoscopy as initial test

Abnormal mt-sDNA test	Colonoscopy after abnormal mt-sDNA *n* (%)	Colonoscopy as initial screening test *n* (%)
	57/254 (22.4)	NA
Mt-sDNA follow-up colonoscopy adherence	49/57 (86.0)	NA
Adequate bowel preparation	49/49 (100.0)	257/279 (93.1)
Cecum reached	49/49 (100.0)	274/279 (99.3)
Biopsy collected	45/49 (91.8)	195/279 (70.7)
Colonoscopy Histology		
Category 1: Stage I-IV colorectal cancer, any size	3/49 (6.1)	6/276 (2.2)
Category 2: Advanced Precancerous Lesions (APL), including the following subcategories:	26/49 (53.1)	62/276 (22.5)
2.1 High-grade dysplasia or ≥ 10 adenomas, any size	0/49 (0.0)	3/276 (1.1)
2.1a High-grade dysplasia, any size	0/49 (0.0)	2/276 (0.7)
2.1b ≥ 10 adenomas, any size	0/49 (0.0)	1/276 (0.4)
2.2 Tubulovillous adenoma, any size	4/49 (8.2)	14/276 (5.1)
2.3 Tubular Adenoma ≥ 10 mm	12/49 (24.5)	42/276 (15.2)
2.4 Sessile serrated lesions with dysplasia (SSLDs); Traditional serrated adenoma (TSA); Conventional adenomas with serrated architecture; Sessile serrated lesions, ≥ 10 mm	10/49 (20.4)	5/276 (1.8)
Category 3: 3–9 adenomas or sessile serrated lesions, < 10 mm, non-advanced	3/49 (6.1)	23/276 (8.3)
Category 4: 1–2 adenomas or sessile serrated lesions, 5–9 mm, non-advanced	5/49 (10.2)	41/276 (14.9)
Category 5: 1–2 adenomas or sessile serrated lesions, < 5 mm, non-advanced	5/49 (10.2)	31/276 (11.2)
Category 6: Negative: no adenocarcinoma of the colorectum, no adenomas or sessile serrated lesions	7/49 (14.3)	110/276 (39.9)
6.1 Hyperplastic polyps or non-neoplastic lesions	0/49 (0.0)	13/276 (4.7)
6.2 No lesions on colonoscopy	7/49 (14.3)	97/276 (35.1)
X Index Lesion could not be categorized because tissue/report was lost/not provided or histopathological diagnosis could not be determined.	0/49 (0.0)	1/276 (0.4)

*Abbreviations*: *CRC *Colorectal cancer, *mt-sDNA *multi-target stool DNA, *NA *Not applicable

There was no significant difference between intervention arms on colonoscopy quality measures, and colonoscopy quality was high either after an abnormal mt-sDNA test or as the initial screening test, with 93%-100% having adequate bowel preparation and with the cecum reached (Table 3). Colonic biopsies were collected in 45/49 (92%) colonoscopies after abnormal mt-sDNA tests and 195/276 (71%) of colonoscopies completed as the initial screening test.

Advanced colorectal neoplasms (CRC or advanced pre-cancers) were found in 29/49 (59%) participants with an abnormal mt-sDNA and 68/276 (25%) with colonoscopy as their initial screening test (Table 3). An additional 13/49 (27%) people with an abnormal mt-sDNA test and 95/276 (34%) people who had completed colonoscopy as their initial screening test had non-advanced lesions, and 7/49 (14%) people with an abnormal mt-sDNA test and 110/276 (40%) people who had completed colonoscopy as their initial screening test had hyperplastic polyps or no lesions found on the exam.

## Discussion

Consistent with our hypothesis, this cluster-randomized clinical trial among Alaska Native peoples showed that both a medium-intensity mailed education intervention and a high-intensity patient navigator-facilitated intervention were effective at increasing screening initiation and completion rates compared with usual care, with screening rates 4.9 times higher among the medium-intensity intervention group and 5.9 times higher among the high-intensity intervention group compared with usual care. Fewer people overall were able to be reached for the intervention in the medium-intensity group, but the effect size between the two intervention groups was not statistically significant nor large enough to be clinically meaningful, suggesting that mailed health education with a follow-up phone call may be a practical approach to increasing screening in primary healthcare delivery systems with limited program resources. The intervention was also effective for both men and women, as well as all age groups.

The Alaska Tribal Health System operates as a “spoke and hub” model, with small community clinics staffed by community health aides supported by advanced practice providers and medical doctors based at regional hospitals. This model is heavily reliant on primary care for cancer prevention and to help detect illness earlier, as well as chronic disease management. Our intervention results show that population-based outreach can be used in communities to help increase CRC screening, which has implications for other screenable cancers as well. Further research to understand patterns of cancer screening referrals and integrating different screening methods into primary care settings within the Alaska Tribal Health System, as well as other health systems, would be helpful.

Of note, 38% of the study population were already screened prior to the start of the intervention, which means that the intervention was able to increase CRC screening in those who might have been exposed to screening promotion messages previously but had not acted to get screened. However, about a quarter (26%) of participants could not be contacted to offer the intervention, representing a missed opportunity, which might be improved by more direct community- or primary care office-visit-based methods [27]. Overall, 26% of the eligible population were screened because of the intervention. If the sample is restricted to contacted individuals, about half (52%) completed screening by any method. A similar percentage overall completed colonoscopy (14%) as had a valid mt-sDNA test (13%). Although encouraging, these results suggest that there may be additional yet unknown barriers that limit screening completion, and more factors that need exploration to increase screening to national benchmarks [28, 29].

Adherence to screening using mt-sDNA was similar in our study (51%) to a national study of Medicaid enrollees which found an overall mt-sDNA adherence of 51% [30], but lower than studies of Medicare beneficiaries, where adherence rates varied between 67% and 88% within 12 months [31–34]. Multiple studies have found that CRC mortality increases if there are delays in colonoscopy follow-up after abnormal stool-based tests [35–38], with follow-up generally recommended within 6–9 months [39]. The abnormal mt-sDNA follow-up to diagnostic colonoscopy rate in our study (86%) was in the middle of the range (72%-96%) of other studies [31, 40, 41]. An additional 5% had follow-up colonoscopies > 18 months after their abnormal mt-sDNA, indicating a need for more timely receipt of diagnostic colonoscopy. Our study showed that about 25% of colonoscopies performed as the initial screening test found CRC/advanced neoplasia, which is slightly lower than other studies within the Alaska Native population which have found 34–67% of colonoscopies detected CRC/advanced neoplasia, with the highest percentages found among those ≥ 50 years old [6, 42]. However, our findings are much higher than national studies of other populations which report about a 6% prevalence of CRC/advanced adenomas in average risk people [43, 44].

Our study used a hospital-based patient navigator to communicate with participants about screening, as patient navigation has been noted in multiple studies to improve screening uptake [45–47]. However, some studies have found that mt-sDNA tests ordered by gastroenterologists have higher adherence than those ordered by primary care or other provider types [30, 32]. It would be worth exploring further which types of providers and healthcare workers might be most effective at encouraging screening behaviors among the Alaska Native population. Additionally, since the patient navigator was locally based, it would be helpful to conduct further research into whether there are differences in mt-sDNA uptake and completion if outreach and instructions are provided instead by Exact Sciences patient navigators located outside of Alaska.

As hypothesized initially, more participants (70%) preferred mt-sDNA for screening compared with colonoscopy; yet limitations in getting samples to the lab in time for analysis meant that almost 40% of kits were unable to be processed in time, primarily due to weather-related postal delays. Midway through the trial, the mt-sDNA sample stability window was extended from 72 to 96 h, and the current mt-sDNA test is viable for up to 144 h, increasing the sample use window even further.

The mt-sDNA positivity rate was 22%, higher than reported in other studies (8%-15%) [31, 34]. Alaska Native people in this intervention had similar findings on mt-sDNA follow-up colonoscopies as reported in other populations, with 59% of people having advanced adenoma or cancer found on follow-up exams, compared with 51–77% in other populations [31, 40]. This demonstrates the potential high utility of mt-sDNA for preventing cancer if test shipment viability issues are lessened.

There were several strengths and limitations of this study. The study sample was derived from a real-world tribal health system population for whom complete medical records and screening status were available, including prior history of CRC screening, family history of CRC, or previous abnormal colonoscopy findings, such that people who were at average risk and eligible for mt-sDNA could be ascertained prior to the trial start. The use of cluster randomization at the community level reduced contamination between screening arms, and the study was conducted in a population not traditionally included in U.S.-based studies. Additionally, all screening services, including colonoscopy, were offered without cost to participants to decrease financial barriers to screening. However, some costs related to colonoscopy including travel for the person and their medical escort, were not always covered, which might have reduced participant interest in colonoscopy. Additionally, the sample included only Alaska Native people from one area of Alaska served by a single healthcare system, so the observed effectiveness of the intervention might not be generalizable to other populations, urban areas, tribal groups, or clinical settings. We also did not measure social elements of health characteristics that might have affected screening participation, including education, income, and language. As the intervention was conducted by telephone and mail, we excluded people without contact information or who had not visited the clinic within three years, thus excluding people who may use healthcare less frequently. More information is needed on test preferences and screening rates among those who do not seek routine healthcare or have other barriers to access. The study also used a patient navigator who was employed by the regional tribal health organization and had worked many years at the hospital, so was familiar with the regional setting in a way that Exact Sciences navigators would not have been using the commercial Cologuard process. The study also did not test colonoscopy and mt-sDNA against FIT for feasibility, which would be helpful as FIT starts to be used more in the Alaska primary healthcare setting. Lastly, the trial was conducted during the COVID-19 pandemic in which elective procedures were postponed. This greatly reduced colonoscopy capacity and increased wait times. Therefore, the observed preference for mt-sDNA seen in our study might have been influenced by this clinical milieu, and preferences might vary if capacity constraints had been less pronounced.

## Conclusions

In this cluster-randomized trial, use of patient navigation and mailed outreach significantly increased Alaska Native CRC screening compared with usual care, with mt-sDNA preferred over colonoscopy. Importantly, the medium-intensity intervention—consisting of a single mailed letter and follow-up phone call—achieved screening rates comparable to the more resource-intensive high-intensity approach in this geographically isolated population. This suggests that mailed outreach with a follow-up phone call may be a scalable and cost-effective strategy for health systems with limited infrastructure. These findings highlight the clinical utility of focused outreach strategies and support the use of mt-sDNA as a viable alternative to colonoscopy in geographically isolated populations. The preference for mt-sDNA and its comparable detection rates for advanced neoplasia further support its integration into routine practice, especially where colonoscopy access is constrained. To ensure sustainability, future implementation efforts should explore adaptations of these interventions across other health systems and rural populations. These findings offer insights into improving CRC screening equity and reducing cancer burden in underserved communities.

## Supplementary Information

Supplementary Material 1.

## Data Availability

The dataset supporting the conclusions of this article is not available due to tribal research data restrictions. Researchers interested in these data may contact the corresponding author for more information on the process for requesting access to these tribal data.

## References

[1] American Cancer Society. Colorectal Cancer Facts & Figs. In. Atlanta, GA: American Cancer Society; 2023. pp. 2023–5.

[2] Zauber AG, Lansdorp-Vogelaar I. Changes in risk factors and increases in screening contribute to the decline in colorectal cancer mortality, 1975 to 2000. Gastroenterology. 2010;139(2):698. 20600066 10.1053/j.gastro.2010.06.011

[3] Haverkamp D, Redwood D, Roik E, Vindigni S, Thomas T. Elevated colorectal cancer incidence among American Indian/Alaska Native persons in Alaska compared to other populations worldwide. Int J Circumpolar Health. 2023;82(1):2184749. 36867106 10.1080/22423982.2023.2184749 PMC9987760

[4] AlaskaDepartment of Health and Social Services. Alaska Behavioral Risk Factor Surveillance System Data Center. Alaska Department of Health and Social Services, Juneau, Alaska; 2024. https://alaska-dph.shinyapps.io/BRFSS/.

[5] Summary, File. 1, Tables PCT1, PCT2, PCT3. Race Reporting for the American Indian and Alaska Native Population by Selected Tribes: 2010 [http://factfinder2.census.gov/faces/nav/jsf/pages/index.xhtml].

[6] Nash SH, Verhage E, Flanagan C, Haverkamp D, Roik E, Zimpelman G, Redwood D. Clinical Outcomes from the Alaska Native Tribal Health Consortium Colorectal Cancer Control Program: 2009–2015. Int J Environ Res Public Health. 2024;21(5). 10.3390/ijerph21050552. 10.3390/ijerph21050552 PMC11120796 38791767

[7] Imperiale TF, Ransohoff DF, Itzkowitz SH, Levin TR, Lavin P, Lidgard GP, Ahlquist DA, Berger BM. Multitarget stool DNA testing for colorectal-cancer screening. N Engl J Med. 2014;370(14):1287–97. 24645800 10.1056/NEJMoa1311194

[8] Heigh RI, Yab TC, Taylor WR, Hussain FT, Smyrk TC, Mahoney DW, Domanico MJ, Berger BM, Lidgard GP, Ahlquist DA. Detection of colorectal serrated polyps by stool DNA testing: comparison with fecal immunochemical testing for occult blood (FIT). PLoS ONE. 2014;9(1):e85659. 24465639 10.1371/journal.pone.0085659 PMC3896420

[9] Seeff LC, Major A, Townsend JS, Provost E, Redwood D, Espey D, Dwyer D, Villanueva R, Larsen L, Rowley K, et al. Comprehensive cancer control programs and coalitions: partnering to launch successful colorectal cancer screening initiatives. Cancer Causes Control. 2010;21(12):2023–31. 21086035 10.1007/s10552-010-9664-9

[10] Donaldson EA, Holtgrave DR, Duffin RA, Feltner F, Funderburk W, Freeman HP. Patient navigation for breast and colorectal cancer in 3 community hospital settings: an economic evaluation. Cancer. 2012;118(19):4851–9. 22392629 10.1002/cncr.27487

[11] Freeman HP. The origin, evolution, and principles of patient navigation. Cancer Epidemiol Biomarkers Prev. 2012;21(10):1614–7. 23045534 10.1158/1055-9965.EPI-12-0982

[12] The Guide to Community Preventive Services. Cancer Prevention and Control: Increasing breast, cervical, and colorectal cancer screening [http://www.thecommunityguide.org/cancer/index.html].

[13] Community Preventive Services Task F. Updated recommendations for client- and provider-oriented interventions to increase breast, cervical, and colorectal cancer screening. Am J Prev Med. 2012;43(1):92–6. 22704753 10.1016/j.amepre.2012.04.008

[14] Ylitalo KR, Camp BG, Umstattd Meyer MR, Barron LA, Benavidez G, Hess B, Laschober R, Griggs JO. Barriers and Facilitators of Colorectal Cancer Screening in a Federally Qualified Health Center (FQHC). J Am Board Fam Med. 2019;32(2):180–90. 30850454 10.3122/jabfm.2019.02.180205

[15] Flanagan CA, Finney Rutten LJ, Kisiel JB, Lent JK, Bachtold JF, Swartz AW, Redwood DG. Development of a colorectal cancer screening intervention for Alaska Native people during a pandemic year. Contemp Clin Trials Commun. 2022;30:101016. 36276262 10.1016/j.conctc.2022.101016 PMC9579295

[16] Glasgow RE, Vogt TM, Boles SM. Evaluating the public health impact of health promotion interventions: the RE-AIM framework. Am J Public Health. 1999;89(9):1322–7. 10474547 10.2105/ajph.89.9.1322 PMC1508772

[17] Tariq S, Woodman J. Using mixed methods in health research. JRSM short Rep. 2013;4(6):2042533313479197. 23885291 10.1177/2042533313479197 PMC3697857

[18] Ivankova NV, Creswell JW, Stick SL. Using mixed-methods sequential explanatory design: from theory to practice. Field Methods. 2006;18:3–20.

[19] Dickerson D, Baldwin JA, Belcourt A, Belone L, Gittelsohn J, Keawe’aimoku Kaholokula J, Lowe J, Patten CA, Wallerstein N. Encompassing Cultural Contexts Within Scientific Research Methodologies in the Development of Health Promotion Interventions. Prev Sci 2018. 10.1007/s11121-018-0926-1 PMC6311146 29959716

[20] Israel BA, Eng E, Schulz AJ, Parker EA, editors. Methods in community-based participatory research for health. San Francisco, CA: Jossey-Bass; 2005.

[21] Frontier. and Remote Area Codes [https://www.ers.usda.gov].

[22] Harris PA, Taylor R, Thielke R, Payne J, Gonzalez N, Conde JG. Research electronic data capture (REDCap)--a metadata-driven methodology and workflow process for providing translational research informatics support. J Biomed Inform. 2009;42(2):377–81. 18929686 10.1016/j.jbi.2008.08.010 PMC2700030

[23] Exact Sciences Corporation. FDA Summary of Safety and Effectiveness Data for the Multi-Target Stool DNA (mt-sDNA) Based Colorectal Cancer Screening Test. In.; 2024: 24.

[24] Alaska Department of Health and Social Services. In: Juneau, editor. Alaska Behavioral Risk Factor Surveillance System. AK: Alaska Department of Health and Social Services; 2020.

[25] Molero-Calafell J, Buron A, Castells X, Porta M. Intention to treat and per protocol analyses: differences and similarities. J Clin Epidemiol. 2024;173:111457. 38977160 10.1016/j.jclinepi.2024.111457

[26] R Core Team: R: A language and environment for statistical computing. In, Vienna. Austria: R Foundation for Statistical Computing; 2021.

[27] Shepherd ME, Lecorps A, Harris-Shapiro J, Miller-Wilson LA. Evaluating Outreach Methods for Multi-Target Stool DNA Test for Colorectal Cancer Screening Among an Employer Population. J Prim care community health. 2021;12:21501327211037892. 34382887 10.1177/21501327211037892 PMC8366118

[28] Zhu X, Weiser E, Griffin JM, Limburg PJ, Finney Rutten LJ. Factors influencing colorectal cancer screening decision-making among average-risk US adults. Prev Med Rep. 2022;30:102047. 36531086 10.1016/j.pmedr.2022.102047 PMC9747631

[29] 80%. in Every Community Strategic Plan [https://nccrt.org ].

[30] Miller-Wilson LA, Finney Rutten LJ, Van Thomme J, Burak Ozbay A, Laffin J, Limburg P. Cross-sectional adherence with the multi-target stool DNA test for colorectal cancer screening in a medicaid population. Prev Med Rep. 2022;30:102032. 36531112 10.1016/j.pmedr.2022.102032 PMC9747618

[31] Prince M, Lester L, Chiniwala R, Berger B. Multitarget stool DNA tests increases colorectal cancer screening among previously noncompliant Medicare patients. World J Gastroenterol. 2017;23(3):464–71. 28210082 10.3748/wjg.v23.i3.464 PMC5291851

[32] Weiser E, Parks PD, Swartz RK, Thomme JV, Lavin PT, Limburg P, Berger BM. Cross-sectional adherence with the multi-target stool DNA test for colorectal cancer screening: Real-world data from a large cohort of older adults. J Med Screen. 2021;28(1):18–24. 32054393 10.1177/0969141320903756 PMC7905742

[33] Miller-Wilson LA, Rutten LJF, Van Thomme J, Ozbay AB, Limburg PJ. Cross-sectional adherence with the multi-target stool DNA test for colorectal cancer screening in a large, nationally insured cohort. Int J Colorectal Dis. 2021;36(11):2471–80. 34019124 10.1007/s00384-021-03956-0 PMC8138513

[34] Strelow B, Dorff H, Gunneson C, Katula W, Pokuta J, Vang K, Olson R, O’Laughlin D. Reducing barriers to colorectal cancer screening via at-home multitarget stool DNA tests. JAAPA. 2025;38(5):42–3. 40273164 10.1097/01.JAA.0000000000000108

[35] San Miguel Y, Demb J, Martinez ME, Gupta S, May FP. Time to Colonoscopy After Abnormal Stool-Based Screening and Risk for Colorectal Cancer Incidence and Mortality. Gastroenterology. 2021;160(6):1997–2005. e1993. 33545140 10.1053/j.gastro.2021.01.219 PMC8096663

[36] Corley DA, Jensen CD, Quinn VP, Doubeni CA, Zauber AG, Lee JK, Schottinger JE, Marks AR, Zhao WK, Ghai NR, et al. Association Between Time to Colonoscopy After a Positive Fecal Test Result and Risk of Colorectal Cancer and Cancer Stage at Diagnosis. JAMA. 2017;317(16):1631–41. 28444278 10.1001/jama.2017.3634 PMC6343838

[37] Beshara A, Ahoroni M, Comanester D, Vilkin A, Boltin D, Dotan I, Niv Y, Cohen AD, Levi Z. Association between time to colonoscopy after a positive guaiac fecal test result and risk of colorectal cancer and advanced stage disease at diagnosis. Int J Cancer. 2020;146(6):1532–40. 31173655 10.1002/ijc.32497

[38] Lee YC, Fann JC, Chiang TH, Chuang SL, Chen SL, Chiu HM, Yen AM, Chiu SY, Hsu CY, Hsu WF, et al. Time to Colonoscopy and Risk of Colorectal Cancer in Patients With Positive Results From Fecal Immunochemical Tests. Clin Gastroenterol Hepatol. 2019;17(7):1332–40. e1333. 30391435 10.1016/j.cgh.2018.10.041

[39] Robertson DJ, Lee JK, Boland CR, Dominitz JA, Giardiello FM, Johnson DA, Kaltenbach T, Lieberman D, Levin TR, Rex DK. Recommendations on Fecal Immunochemical Testing to Screen for Colorectal Neoplasia: A Consensus Statement by the US Multi-Society Task Force on Colorectal Cancer. Gastroenterology. 2017;152(5):1217–37. e1213. 27769517 10.1053/j.gastro.2016.08.053

[40] Cooper GS, Grimes A, Werner J, Cao S, Fu P, Stange KC. Barriers to Follow-Up Colonoscopy After Positive FIT or Multitarget Stool DNA Testing. J Am Board Fam Med. 2021;34(1):61–9. 33452083 10.3122/jabfm.2021.01.200345

[41] Chamberlain AM, Ebner DW, Jenkins GD, Greene M, Finney Rutten LJ. Follow-up Colorectal Cancer Screening After Negative-Result and Positive-Result Multitarget Stool DNA Tests: A Population-Based Study in Southeast Minnesota. Mayo Clin Proc Innov Qual Outcomes. 2025;9(2):100599. 40092493 10.1016/j.mayocpiqo.2025.100599 PMC11909756

[42] Redwood DG, Prewitt JJ, Holt MC, Gerrish SS. Elevated Adenomatous Polyp Detection Rate Among Alaska Native and American Indian People in Interior Alaska, 2018–2022. Public Health Rep 2023:333549221143204. 10.1177/00333549221143204 PMC10515984 36683459

[43] Heitman SJ, Ronksley PE, Hilsden RJ, Manns BJ, Rostom A, Hemmelgarn BR. Prevalence of adenomas and colorectal cancer in average risk individuals: a systematic review and meta-analysis. Clin Gastroenterol Hepatol. 2009;7(12):1272–8. 19523536 10.1016/j.cgh.2009.05.032

[44] Imperiale TF, Abhyankar PR, Stump TE, Emmett TW. Prevalence of Advanced, Precancerous Colorectal Neoplasms in Black and White Populations: A Systematic Review and Meta-analysis. Gastroenterology. 2018;155(6):1776–86. e1771. 30142339 10.1053/j.gastro.2018.08.020

[45] Rice K, Sharma K, Li C, Butterly L, Gersten J, DeGroff A. Cost-effectiveness of a patient navigation intervention to increase colonoscopy screening among low-income adults in New Hampshire. Cancer. 2019;125(4):601–9. 30548480 10.1002/cncr.31864 PMC6399743

[46] Freeman HP. Patient navigation: a community centered approach to reducing cancer mortality. J Cancer Educ. 2006;21(1 Suppl):S11–14. 17020496 10.1207/s15430154jce2101s_4

[47] Elkin EB, Shapiro E, Snow JG, Zauber AG, Krauskopf MS. The economic impact of a patient navigator program to increase screening colonoscopy. Cancer. 2012;118(23):5982–8. 22605672 10.1002/cncr.27595

