# Supplementary material for: Increasing colorectal cancer screening among Alaska Native peoples living in remote areas of Alaska: a multitarget stool DNA cluster randomized controlled trial
Source: BMC Prim Care. 2026 May 26;27:303. doi: 10.1186/s12875-026-03370-x (PMC13471518; doi:10.1186/s12875-026-03370-x)
Supplement: Supplementary file 1 [file 12875_2026_3370_MOESM1_ESM.docx]

| **Supplemental eTable 1. Multi-target stool DNA (mt-sDNA) or colonoscopy screening test selection, by age and sex.** | | | | | | | | |
| --- | --- | --- | --- | --- | --- | --- | --- | --- |
|  | **Test Selected, n (%)** | | | | | | | |
|  | mt-sDNA (n=661) | | | Colonoscopy (n=288) | | | | *P* value |
| By Age |  | | |  | | | | <0.01 |
| 45-54 years | 280/441 (63.5) | | | 161/441 (36.5) | | | |  |
| 55-64 years | 239/328 (72.9) | | | 89/328 (27.1) | | | |  |
| 65-75 years | 142/180 (78.9) | | | 38/179 (21.1) | | | |  |
| By Sex |  | | |  | | | | 0.26 |
| Women | 285/419 (68.0) | | | 134/419 (32.0) | | | |  |
| Men | 376/530 (70.9) | | | 154/530 (29.1) | | | |  |
| **Supplemental eTable 2. Multi-target stool DNA (mt-sDNA) or colonoscopy screening test completion by intervention arm within 6 or 18 months.** | | | | | | | | |
|  | | Total | Usual Care | | Medium Intensity | High Intensity | *P* value | |
| **Overall** | |  |  | |  |  |  | |
| Screening completion within 6 months among total study population | | 311/2029 (15.3) | 21/587 (3.6) | | 124/696 (17.8) | 166/746 (22.3) | <0.01 | |
| Screening completion within 18 months among total study population | | 500/2029 (24.6) | 50/587 (8.5) | | 197/696 (28.3) | 253/746 (33.9) | <0.01 | |
|  | |  |  | |  |  |  | |
| **By Intervention Arm** | |  |  | |  |  |  | |
| Screening completion within 6 months among those who completed screening | | 290/513 (56.5) | NA | | 124/229 (54.1) | 166/284 (58.5) | 0.62 | |
| Screening completion within 18 months among those who completed screening | | 450/513 (87.7) | NA | | 197/229 (86.0) | 253/284 (89.1) | 0.46 | |
|  | |  |  | |  |  |  | |
| Colonoscopy completion within 6 months among total colonoscopies completed | | 42/240 (17.5) | NA | | 22/118 (18.6) | 20/122 (16.4) | 0.53 | |
| Colonoscopy completion within 18 months of total colonoscopies completed | | 172/240 (71.5) | NA | | 84/118 (71.2) | 88/122 (72.1) | 0.87 | |
|  | |  |  | |  |  |  | |
| Mt-sDNA completion within 6 months of total mt-sDNA tests completed | | 235/254 (92.5) | NA | | 94/100 (94.0) | 141/154 (91.6) | 0.51 | |
| Mt-sDNA completion within 18 months of total mt-sDNA tests completed | | 254/254 (100) | NA | | 100/100 (100) | 154/154 (100) | NA | |

| **Supplemental eTable 3. Multi-target stool DNA (mt-sDNA), colonoscopy, and fecal immunochemical test (FIT) screening test completion, by age and sex.** | | | | |
| --- | --- | --- | --- | --- |
|  | **Test Completed, n (%)** | | | |
|  | mt-sDNA | Colonoscopy | FIT | *P* value |
| By Age |  |  |  |  |
| 45-54 years | 114/262 (43.5) | 128/262 (48.9) | 20/262 (7.6) | <0.01 |
| 55-64 years | 83/195 (42.6) | 84/195 (43.1) | 28/195 (14.4) |  |
| 65-75 years | 55/110 (50.0) | 29/110 (26.4) | 26/100 (23.6) |  |
|  |  |  |  |  |
| By Sex |  |  |  |  |
| Women | 97/259 (37.5) | 126/259 (48.6) | 36/259 (13.9) | <0.01 |
| Men | 155/308 (50.3) | 115/308 (37.3) | 38/308 (12.3) |  |
